# Molecular insights into vascular aging

**DOI:** 10.18632/aging.101697

**Published:** 2018-12-05

**Authors:** Kirsty Foote, Martin R. Bennett

**Affiliations:** 1 Division of Cardiovascular Medicine, University of Cambridge, Addenbrooke’s Centre for Clinical Investigation, Addenbrooke’s Hospital, Cambridge, UK

**Keywords:** aging, mitochondria, vascular stiffness

Aging of the vasculature is the leading risk factor for cardiovascular and cerebrovascular disease [1]. As such, strategies that delay vascular aging could minimize the risk of developing life-threatening vascular complications, enhance healthspan and prolong lifespan. However, such preventative and therapeutic approaches require identification of the pathophysiological changes underlying vascular aging, and the associated molecular mechanisms responsible.

The healthy artery comprises endothelial cells (ECs), vascular smooth muscle cells (VSMCs) and the extracellular matrix (ECM), all of which are susceptible to damage or disruption during aging [2]. The intima is composed of ECs, their basement membrane, and (in humans) occasional VSMCs. ECs line the lumen, act as a selective permeability barrier and anticoagulant surface, and EC function helps control vascular tone. VSMCs form the bulk of the media and are primarily responsible for contraction and relaxation of the vessel. VSMCs also undergo proliferation, migration and de-differentiation during vascular remodeling in injury or disease. The ECM is composed of structural proteins such as collagens and elastin that tether VSMCs together, provides structural support, and regulates the mechanical function of the vessel. Aging is associated with changes in both cellular components and the ECM. For example, aging induces EC dysfunction, reducing barrier integrity, promoting inflammation, and reducing the capacity of the vessel to vasodilate. VSMCs de-differentiate from a contractile to a synthetic phenotype, where they become pro-migratory and proliferative, and can undergo apoptosis and senescence. Aging is associated with increased deposition of collagen and breakages in the elastic laminae. The overall effects of aging lead to increased arterial stiffness and inflammation, thicker walls and lumen dilation, and a diminished ability to contract and relax.

**Figure 1:**
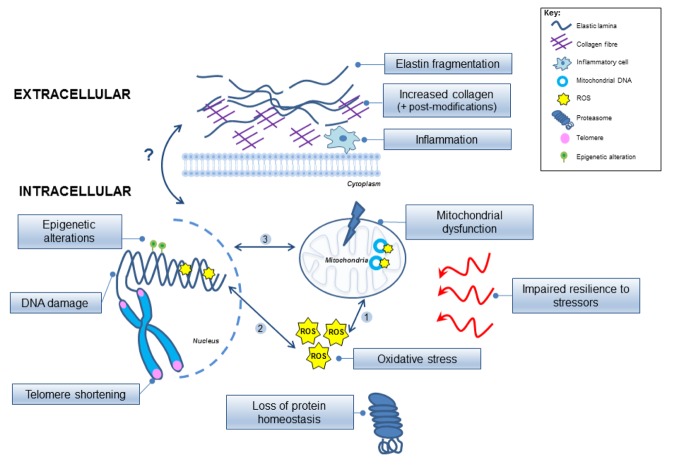
**Model implicating the intracellular and extracellular molecular mechanisms involved in vascular aging.** The inter-relationships between the processes remain unclear and are depicted by arrows highlighting possible links. (1) Reactive oxygen species (ROS) can damage the mitochondria and mitochondrial DNA, which can further exacerbate mitochondrial dysfunction, and (2) can also damage the nuclear genome (yellow stars); (3) Inter-communication is evident between the mitochondrial and nuclear genomes but remains complex. Epigenetic modifications are also involved in altering the nuclear DNA (green circles), and the chromosomal telomeres become shortened. Protein homeostasis is disrupted, and overall cellular resilience to molecular stressors (red arrows) is impaired. ROS, reactive oxygen species.

Rapid advances in geroscience have identified a ‘Molecular Signature’ for the aged vessel that represents distinct underlying intracellular and extracellular molecular processes (Figure 1). Intracellular events involve: oxidative stress, mitochondrial dysfunction, cellular senescence, DNA damage, epigenetic changes, telomere shortening, loss of proteostasis and an impaired resilience to stressful stimuli. Extracellular events include inflammatory cell infiltration, and largely involve the ECM, including alterations in collagen/elastin content and post-synthetic modifications to collagen such as enhanced cross-linking.

Although all of these processes are associated with vascular aging, it is unclear whether they are a cause or consequence of aging, how they are inter-related, and whether targeting one or more is sufficient to prevent or delay vascular aging. For example, we recently reported that mitochondrial function progressively deteriorates with aging of mouse arteries, associated with reduced mitochondrial DNA copy number (mtCN) and expression of enzymes that regulate mitochondrial DNA synthesis; we showed that increasing mtCN enhanced mitochondrial function and delayed the onset of multiple structural and functional parameters of vascular aging [3]. Although this demonstrates that genetic augmentation of mitochondrial function delays vascular aging, the mechanisms underlying mitochondrial dysfunction and failure of the nuclear genome to compensate for reduced mitochondrial function and increase mtCN are not known. Reduced mtCN was associated with up-regulation of Twinkle helicase and transcription factor A, mitochondrial (TFAM), two major nuclear-encoded regulators of mtCN, and other nuclear-encoded proteins involved in mitochondrial function including peroxisome proliferator-activated receptor gamma coactivator 1-alpha (PGC1α) and complex II of the electron transport chain. However, the failure of this compensatory communicative response between the two cellular genomes to restore mtCN levels with age raises the possibility that the inability to repair the aging deficit in mtCN may be irreversible beyond a certain point.

In contrast, the ability of enhanced mitochondrial function since birth (as in our genetically altered mice) to delay vascular decline suggests that preventative and early intervention are key to delay vascular aging. Indeed, several studies have demonstrated beneficial effects of long-standing lifestyle changes such as caloric restriction (CR) and exercise, and compounds that mimic these processes. For example, β-hydroxybutyrate, a ketone released naturally during CR, enhances cell division and protects against DNA damage and cellular senescence in ECs and VSMCs in older fasted mice via the Oct4-mediated LaminB1 pathway [4]. Similarly, the nicotinamide adenine dinucleotide (NAD^+^) booster nicotinamide mononucleotide (NMN) promoted angiogenesis and recapitulated the benefits of exercise via sirtuin 1-mediated inhibition of Notch signalling in aged exercised mice [5].

Vascular stiffness is a major link between normal vascular aging and development of vascular complications. Although the marked changes in ECM content and structure in aging influence vascular mechanical properties directly, aging also affects cell-ECM interactions, and the stiffness of VSMCs themselves. For example, VSMCs can take on a stiffness phenotype [6] dependent on their differentiation and the local molecular environment. Although the precise links between VSMC stiffness and altered contractility are not fully understood, VSMC stiffness appears to be regulated through cell-cell or cell-ECM interconnectivity through cell surface receptors, which also control a host of signalling pathways that regulate VSMC proliferation and differentiation [2].

In summary, identification of the distinct molecular pathways involved in vascular aging provides great potential for therapeutics. Research efforts should focus on disentangling the complex interactions between the molecular processes involved, and testing the feasibility and timing of administration of potential therapeutics that can limit the effects of oxidative stress, mitochondrial dysfunction, cellular senescence, and DNA damage. Arterial stiffness and central blood pressure (cBP) are excellent predictors of age-related complications [7] and can identify individuals at high risk allowing targeting of treatments. Rejuvenating the vascular wall may supplement lifestyle modifications such as exercise and CR, with agents that allow these benefits to be continued in later life.
